# Parasocial and interpersonal interaction: distinct associations with customer value co-creation in virtual brand communities

**DOI:** 10.3389/fpsyg.2026.1975666

**Published:** 2026-10-21

**Authors:** Xiaomei Wu, Tianmin Ma, Kaile Wang, Hongyan Lin

**Affiliations:** 1 School of Economics and Management, Xiamen University of Technology, Xiamen, China; 2 College of Natural Sciences, The University of Texas at Austin, Austin, TX, United States

**Keywords:** community identification, cross-sectional mediation, customer value co-creation, interpersonal interaction, media psychology, parasocial interaction, self-efficacy, virtual brand community

## Abstract

Virtual brand communities expose members to communication from the brand side and interaction with other members, but these sources may not relate to participation through the same psychological processes. Drawing on the stimulus organism response framework, social identity theory, and social cognitive theory, this study tested whether community identification and self-efficacy statistically accounted for indirect associations between parasocial interaction, interpersonal interaction, and customer value co-creation behavior. The hypotheses were examined in an unpublished cross-sectional survey of 240 adult users of virtual brand communities in China. An audit of the questionnaire and data codebook corrected a prior item-block parsing error and established the intended 30-item measurement structure. Internal consistency was acceptable for the five composites (Cronbach's alpha = 0.730–0.837; McDonald's omega = 0.744–0.838). A five-factor confirmatory model fit better than models combining the interaction constructs, combining the mediators, or placing all items on one factor, although its absolute fit and discriminant validity were only partly satisfactory (CFI = 0.870, TLI = 0.857, RMSEA = 0.064, SRMR = 0.056). Regression estimates used HC3 robust standard errors, and indirect associations used 5,000 percentile-bootstrap samples. Parasocial interaction uniquely predicted community identification and self-efficacy, and both variables were associated with customer value co-creation. The indirect associations of parasocial interaction through community identification (*B* = 0.163, 95% CI [0.038, 0.303]) and self-efficacy (*B* = 0.167, 95% CI [0.053, 0.281]) were statistically detectable. Interpersonal interaction retained a direct association with customer value co-creation but did not uniquely predict either proposed mediator when both interaction variables were included; its two specific indirect associations were not statistically detectable. Results were similar after excluding uniform responders and after adding demographic and community-use controls. The findings distinguish relational and capability-related correlates of parasocial interaction while showing that interpersonal interaction may share substantial variance with brand-side relational experience. Because the data are cross-sectional, same-source, and affected by incomplete discriminant validity, the indirect effects are interpreted as covariance patterns consistent with the proposed pathways rather than evidence of causal psychological mechanisms.

## Introduction

Social media and mobile communication have transformed virtual brand communities into persistent social environments. Members encounter official brand posts, responses from community operators, peer discussions, product-use stories, and invitations to contribute ideas. These environments are relevant to media psychology because members move between audience-like exposure, relational engagement, peer exchange, and active participation. Customer value co-creation is one form of that participation. It includes activities through which customers provide information, give feedback, recommend the community, help other members, and support service or product improvement (Prahalad and Ramaswamy, 2004; Vargo and Lusch, 2004; Yi and Gong, 2013).

Interaction in virtual brand communities has at least two social sources. The first is the brand side, represented by official accounts, brand messages, and community operators. Repeated exposure to relational brand cues can make an organization appear accessible, friendly, responsive, and socially present. Research has extended parasocial concepts from traditional media figures to social media personalities and consumer-brand relationships (Labrecque, 2014; Lou, 2022). Recent studies also show that social presence and parasocial experience can relate to self-efficacy, persuasion, and behavioral intentions in social media settings (Kim, 2022; Balaban et al., 2022). The second source is other community members. Their exchange of product information, usage experience, advice, and emotional support forms a horizontal peer environment in which members can learn, compare experiences, receive help, and establish reciprocal ties.

The term parasocial interaction requires particular care in interactive platforms. Traditional parasocial interaction describes a psychologically meaningful but structurally asymmetric experience with a mediated persona. Social media complicates that boundary because official accounts may reply, community managers may communicate directly, and members may perceive a continuing relationship even when most contact remains mediated and one-to-many. Lou (2022) describes contemporary influencer-follower relations as potentially trans-parasocial because mediated familiarity and limited reciprocal communication coexist. In the present study, parasocial interaction does not refer to friendship with a named spokesperson or to verified one-to-one communication. It refers to a member's relational perception of the brand side as attractive, trustworthy, friendly, helpful, worth following, and sufficiently accessible to approach. This operationalization therefore overlaps conceptually with perceived interactivity, brand responsiveness, social presence, and consumer-brand relational experience. That boundary is examined empirically rather than assumed to be clean.

Interpersonal interaction has a different target. It concerns exchanges with other members, including product-related communication, experience sharing, emotional exchange, and advice. Such exchanges can provide social support and make community resources visible. Nambisan and Baron (2009) showed that cognitive, social-integration, personal-integration, and hedonic benefits are important in voluntary participation in virtual customer environments. More recent research links social capital and member inspiration to value co-creation in online brand communities (Cao et al., 2022), while digital-affordance research shows that support emerges from the relation between user goals, community responses, and platform features (Kuckertz et al., 2022). Qualitative evidence from luxury fashion brand communities further shows that user-generated content can communicate experiential and symbolic benefits and shape perceived brand value, while also creating context-specific tensions between inclusivity and exclusivity (Basile et al., 2024). Together, these studies suggest that communication source and platform context matter, even when brand-side and peer interaction are correlated.

A direct association between interaction and co-creation does not specify what members experience psychologically. The stimulus organism response framework provides an organizing structure: features of the communication environment can be treated as stimuli, internal cognitive and affective states as organismic responses, and participatory behavior as the response (Mehrabian and Russell, 1974). In this study, parasocial interaction and interpersonal interaction are treated as source-specific social stimuli. Community identification and self-efficacy represent different internal states, and customer value co-creation represents the participatory outcome. The framework organizes the model, but the theoretical content of the mediators comes from social identity theory and social cognitive theory.

Community identification represents a relational pathway. Social identity theory holds that part of the self-concept derives from group membership and from the value and emotional meaning attached to that membership (Tajfel and Turner, 1986). In brand communities, identification includes cognitive awareness of membership, affective attachment, and the evaluative meaning of belonging (Algesheimer et al., 2005; Bagozzi and Dholakia, 2006). Interaction may be relevant because brand-side recognition can reduce psychological distance, while peer exchange can provide shared experience, acceptance, and a sense of collective membership. Prior studies associate community interaction with customer identification (Luo et al., 2016) and community identification with engagement, loyalty, and value co-creation (Kaur et al., 2020; Liu et al., 2024).

Identification may also relate to contribution. When a community becomes part of a member's self-definition, providing feedback, sharing information, helping others, and advocating for the community can express and maintain that identity. An experimental study of online social value co-creation found that participation can increase purchase intention through identification with the company (Shi et al., 2022). Research in online travel communities likewise connects interaction, self-identity, social support, community identification, and citizenship behavior (Jiang et al., 2022). These findings support a relational sequence in which interaction covaries with identification and identification covaries with co-creation.

The first hypotheses concern the associations between the two interaction variables and community identification.

H1a: Parasocial interaction positively predicts community identification.
H1b: Interpersonal interaction positively predicts community identification.
H2: Community identification positively predicts customer value co-creation behavior.

If the covariance pattern is consistent with interaction being linked to co-creation through identification, the product of the interaction-identification path and the identification-co-creation path should be positive. The corresponding hypotheses are stated as statistical mediation hypotheses, while recognizing that cross-sectional data cannot establish temporal mediation.

H3a: Community identification mediates the relationship between parasocial interaction and customer value co-creation behavior.
H3b: Community identification mediates the relationship between interpersonal interaction and customer value co-creation behavior.

Self-efficacy represents a capability pathway. It refers to judgments about one's capacity to organize and perform actions required for a desired outcome (Bandura, 1986, 1997). Self-efficacy is context-specific rather than equivalent to general confidence. In a virtual brand community, contribution-related self-efficacy concerns whether members believe they can use community functions, express their views, share product experience, and respond to other members' questions. These beliefs are relevant because online participation requires people to move from observation to public action and to accept the possibility that a suggestion may be ignored or an answer may be incorrect.

Social cognitive theory identifies mastery experience, vicarious experience, social persuasion, and affective states as sources of self-efficacy. Brand-side communication can provide information and feedback showing that member input is understandable or useful. Peer interaction can provide examples, encouragement, and practical assistance. Hsu et al. (2007) linked knowledge-sharing self-efficacy to sharing behavior in virtual communities, and Alves and Mainardes (2017) identified self-efficacy as a personal resource in value co-creation. Kim (2022) further found that social presence and parasocial interaction could relate to self-efficacy in an Instagram setting. These findings make it plausible that both forms of community interaction are associated with contribution-related self-efficacy.

H4a: Parasocial interaction positively predicts self-efficacy.
H4b: Interpersonal interaction positively predicts self-efficacy.

Members with stronger self-efficacy should be more willing to seek and share information, formulate suggestions, participate in community activities, and help other members. Co-creation self-efficacy has also been positioned as a psychological link between experience and co-creation behavior (Hasan et al., 2024). The second group of mediation hypotheses therefore concerns capability beliefs.

H5: Self-efficacy positively predicts customer value co-creation behavior.
H6a: Self-efficacy mediates the relationship between parasocial interaction and customer value co-creation behavior.
H6b: Self-efficacy mediates the relationship between interpersonal interaction and customer value co-creation behavior.

Community identification and self-efficacy are specified as parallel rather than sequential variables. This choice does not imply that they are causally independent. Identification may expose members to social support that improves efficacy, and successful contribution may reinforce identification. A reciprocal or sequential process is plausible. The parallel specification asks a narrower cross-sectional question: does each variable account for unique covariance with customer value co-creation when both are included? This avoids selecting a temporal order that the design cannot identify. Longitudinal or experimental data would be required to compare the plausible sequences.

The central contribution is therefore not the claim that interaction generally predicts participation. Prior research already supports that association. The study instead compares two interaction sources and two psychological correlates in the same model. It asks whether the relational and capability pathways remain distinguishable after their shared variance is taken into account. The analysis also examines whether participation and citizenship dimensions of customer value co-creation produce the same pattern. Figure 1 presents the proposed parallel model. Direct paths from both interaction variables to customer value co-creation are retained for effect decomposition; they are not separate focal hypotheses.

**Figure 1:**
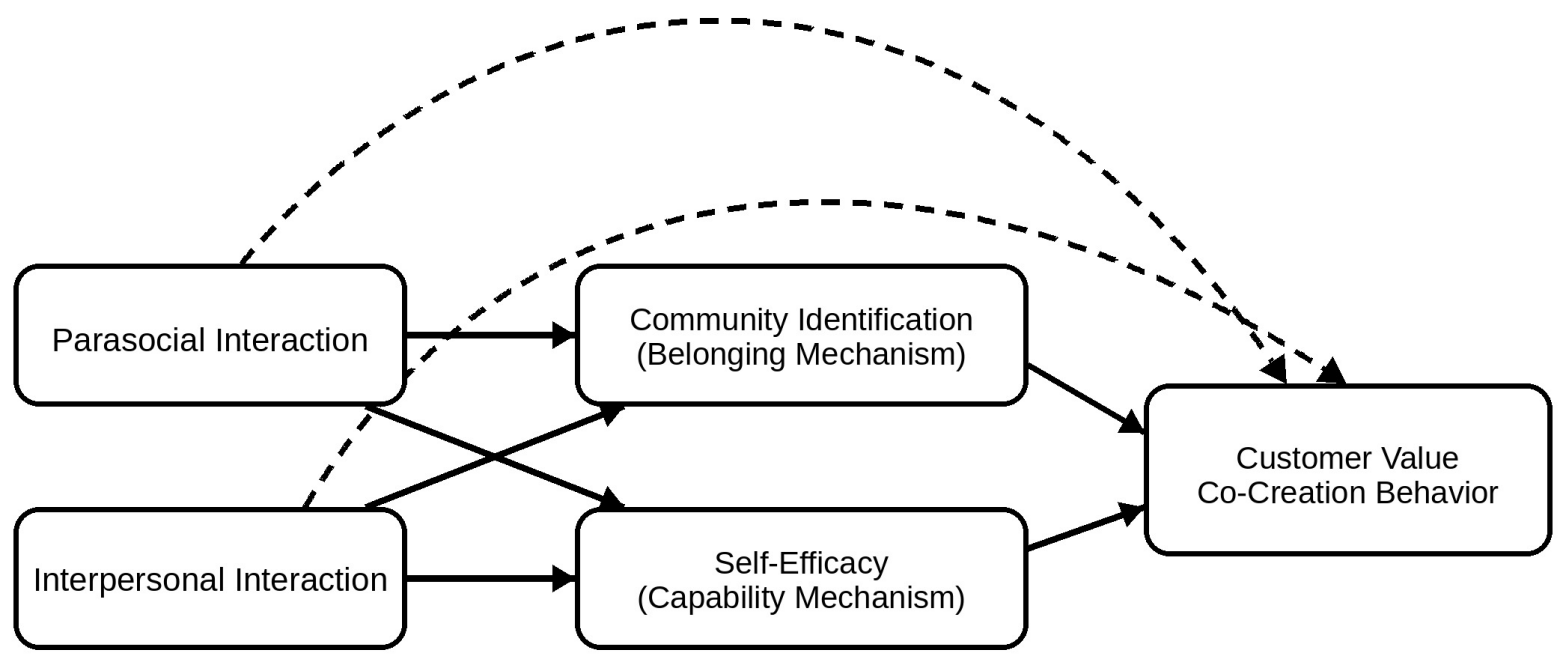
Proposed parallel indirect association model. The arrows represent hypothesized regression paths. Cross-sectional data do not identify temporal or causal direction.

## Materials and methods

### Recruitment and participants

The survey was conducted for an original research project on antecedents of users' value co-creation behavior in virtual brand communities. The online questionnaire was distributed in China through Wenjuanxing between April 20 and May 3, 2022. The link was shared through virtual brand communities and social media using snowball sampling. No monetary or material incentive was offered. The recruitment denominator was not recorded, so an invitation-based response rate cannot be calculated. Eligibility required prior participation in a virtual brand community. Respondents were instructed to answer all focal items with reference to one virtual brand community in which they frequently participated. The communities included official brand communities on social media, brand forums, and fan communities, but the questionnaire did not record a respondent-level platform or community identifier. The analysis therefore cannot model clustering within specific communities or compare community categories.

The raw export contained 240 complete records, with no missing responses among the 30 focal items and no responses outside the permitted range of 1 to 5. All respondents selected an adult age category; 79.2% were between 18 and 30 years of age, 81.7% reported a bachelor's degree, 55.0% were corporate employees, and 30.0% were students. Complete demographic and community-use distributions appear in Table 1.

**Table 1:** Main characteristics of the full analytic sample.

Characteristic	Category	*n*	Percent
Gender	Male	109	45.4
Gender	Female	131	54.6
Age	18 to 25 years	107	44.6
Age	26 to 30 years	83	34.6
Age	31 to 35 years	38	15.8
Age	Over 35 years	12	5.0
Education	High school or below	8	3.3
Education	Associate degree	11	4.6
Education	Bachelor degree	196	81.7
Education	Master degree	22	9.2
Education	Doctorate or above	3	1.3
Monthly income	No income	42	17.5
Monthly income	Below RMB 2,000	17	7.1
Monthly income	RMB 2,000 to 5,000	34	14.2
Monthly income	RMB 5,001 to 10,000	94	39.2
Monthly income	RMB 10,001 to 30,000	42	17.5
Monthly income	RMB 30,001 to 50,000	3	1.3
Monthly income	Above RMB 50,000	8	3.3
Occupation	Student	72	30.0
Occupation	Teacher	4	1.7
Occupation	Government or public institution	19	7.9
Occupation	Corporate employee	132	55.0
Occupation	Self-employed	6	2.5
Occupation	Other	7	2.9
Participation duration	Less than 6 months	47	19.6
Participation duration	6 months to 1 year	50	20.8
Participation duration	1 to 2 years	71	29.6
Participation duration	2 to 3 years	35	14.6
Participation duration	More than 3 years	37	15.4
Visit frequency	Daily	42	17.5
Visit frequency	Every 2 to 4 days	111	46.3
Visit frequency	Once a week	49	20.4
Visit frequency	Twice a month	11	4.6
Visit frequency	Once a month	7	2.9
Visit frequency	Less than once a month	20	8.3

*N* = 240. Percentages may not sum to 100 because of rounding.

The survey platform recorded completion time and network-address metadata. Completion times ranged from 46 to 3,905 seconds, with a median of 221.5 seconds. No preregistered or instrument-validated minimum completion-time cutoff was available, so response time was described but not used for retrospective exclusion. There were 239 unique network addresses among 240 records; the two records sharing one address were retained because a shared address does not establish duplicate participation. No dedicated bot-detection field or attention-check item was available. Fifteen participants selected one identical response option across all 30 focal items. Because all focal items were positively keyed, such a pattern was not logically inconsistent. The full sample of 240 was therefore used for the primary analysis, and exclusion of these 15 uniform responders was treated as a sensitivity analysis rather than a validity rule.

### Ethics and consent

The study used a low-risk online questionnaire under the institutional requirements applicable at Xiamen University of Technology. Prior committee review was not required under those requirements. An institutional ethics-exemption determination was issued retrospectively during manuscript submission after data collection. Before entering the questionnaire, participants viewed a consent screen explaining the academic purpose of the study, voluntary participation, the option to leave the questionnaire, aggregate research use of responses, and confidentiality. Participants who selected agreement proceeded to the questionnaire. No names or contact details were requested. The survey platform automatically recorded network-address metadata. The metadata were used only to inspect potential duplicate submissions, excluded from the analytic variables, and omitted from shared data.

### Measures

All focal items used a five-point response scale from 1 strongly disagree to 5 strongly agree. There were no reverse-coded items. Composite scores were calculated as the mean of their assigned items, with higher values representing higher levels of the named variable. Appendix A provides the complete English wording, the Chinese wording used in the questionnaire, construct assignment, and sources.

Parasocial interaction was measured with 10 items adapted from Hoerner (1999) and Bao et al. (2011). The items assess perceived attractiveness, continued attention, willingness to approach the brand community, friend-like experience, trustworthiness, helpfulness, information sharing, positive affect, friendliness, and perceived value of attention. The target was the brand side as represented in the focal community, not a named influencer, spokesperson, or individual manager. The scale therefore represents a context-specific parasocial and consumer-brand relational perception. It should not be interpreted as a pure measure of a relationship with an identifiable media persona.

Interpersonal interaction was measured with four items adapted from Wang and Ma (2013) and informed by Nambisan and Baron (2009). The items concern exchanging product information, sharing usage experience, emotional exchange, and giving or receiving advice with other community members.

Community identification was measured with four items adapted from Algesheimer et al. (2005). They concern self-categorization as a community member, pride, belonging, and respect associated with membership. One item considered during early instrument development was not included in the final questionnaire because its content was not sufficiently aligned with the construct; the data and all analyses therefore use the four administered items only.

Customer value co-creation behavior was measured with eight items adapted from Yi and Gong (2013). Four items represent participation behavior: seeking information, providing needed information, fulfilling member responsibilities, and communicating effectively with community operators. Four represent citizenship behavior: giving suggestions, recommending the community, helping other members, and tolerating minor product or service problems. The primary analysis uses the overall eight-item composite. The empirical basis for aggregation is evaluated by comparing one-factor and two-factor measurement models, and supplementary models analyze participation and citizenship separately.

Self-efficacy was measured with four items adapted from Hsu et al. (2007) to the virtual brand community context. The items concern confidence in using community functions without guidance, expressing opinions, sharing product experience, and answering other members' questions. The construct is therefore contribution-related self-efficacy rather than generalized self-efficacy.

Most measures were adapted from established English scales or previously used Chinese-language measures. For English-source items, two bilingual researchers independently translated the items into Chinese. The research team compared the versions, resolved discrepancies, and revised wording for the virtual brand community setting. A formal blind back-translation was not conducted. This limitation is reported because translation review cannot provide the same evidence as a documented translation and back-translation protocol.

### Measurement analysis

Item-level confirmatory factor analysis evaluated a correlated five-factor model. Maximum-likelihood estimation was applied to the standardized item covariance matrix. Standardized factor loadings, chi-square, comparative fit index, Tucker-Lewis index, root mean square error of approximation, and standardized root mean square residual are reported. Model-fit cutoffs were treated as descriptive guides rather than mechanical acceptance rules. The five-factor model was compared with a model that combined parasocial interaction and interpersonal interaction, a model that combined community identification and self-efficacy, and a one-factor model.

Internal consistency was evaluated with Cronbach's alpha, mean inter-item correlation, McDonald's omega, and composite reliability. Convergent validity was summarized with average variance extracted. Discriminant validity was evaluated with factor correlations and the heterotrait-monotrait ratio of correlations using 2,000 bootstrap samples (Henseler et al., 2015). Corrected item-total correlations and alpha-if-deleted statistics appear in Appendix B. Consistent with McNeish (2018), alpha was not treated as sufficient evidence of reliability or dimensionality.

The dimensionality of customer value co-creation behavior was evaluated separately by comparing an eight-item one-factor model with a correlated two-factor model distinguishing participation and citizenship. A higher-order factor model with only two first-order factors is not separately identified without additional constraints, so it was not used as evidence for aggregation. The two subdimensions were instead examined as separate outcomes in sensitivity analyses.

Because all focal variables were measured in one questionnaire, Harman's test was not used to claim absence of common-method variance. A one-factor CFA served only as a poor-fit comparison. An additional sensitivity model added an orthogonal equal-loading common method factor to the five trait factors. This constrained model cannot identify every form of method bias, but it provides a more informative diagnostic than the percentage explained by a first exploratory factor. The interpretation gives common-method concerns substantial weight regardless of model comparison results (Podsakoff et al., 2003, 2012).

### Structural and indirect association analysis

The structural hypotheses were tested with observed composite scores because the five-factor measurement model provided only partial support for distinct latent constructs. Presenting a latent structural model as definitive would imply greater measurement separation than the data support. The composite analysis is therefore reported as an exploratory test of the proposed covariance pattern.

Four ordinary least-squares equations were estimated. Community identification was regressed on parasocial interaction and interpersonal interaction. Self-efficacy was regressed on the same predictors. Customer value co-creation behavior was regressed on both interaction variables and both proposed mediators. A total-association model regressed customer value co-creation behavior on the two interaction variables without the mediators. The direct paths remained in the outcome model so that indirect associations were not made conditional on complete mediation.

The primary models did not include covariates. Demographic variables were not part of a prespecified causal adjustment set, and automatic adjustment for all available characteristics can introduce rather than remove bias. Two sensitivity specifications were nevertheless estimated. The first added gender, age, education, income, and occupation as categorical dummy variables. The second additionally added duration of community participation and visit frequency as categorical dummy variables. This coding avoids an equal-interval assumption for ordinal categories and makes the reference structure explicit in Appendix B.

Regression coefficients are reported in unstandardized and standardized form. HC3 heteroskedasticity-robust standard errors and 95% confidence intervals were used for regression paths because the outcome model showed heteroskedasticity. Linearity was examined with the Ramsey RESET test. Multicollinearity was summarized with variance inflation factors. Leverage and Cook's distance were inspected without applying a *post hoc* exclusion threshold. Residual normality was assessed descriptively; the non-parametric bootstrap was used for indirect associations because product terms have non-normal sampling distributions.

Specific indirect associations were calculated as products of unstandardized coefficients. Both proposed mediators were included simultaneously. Five thousand non-parametric case-resampling bootstrap draws generated percentile 95% confidence intervals. An interval excluding zero was treated as evidence that the corresponding indirect association was statistically detectable. Contrasts between the two specific indirect associations were also bootstrapped. A contrast interval including zero is interpreted as failure to detect a difference, not as evidence of equivalence. The final analyses were rerun in Python 3.12.14 using pandas 2.2.3, NumPy 2.3.5, SciPy 1.17.0, and documented analysis routines. The de-identified participant-level data and analysis materials supporting the conclusions are available from the corresponding author upon reasonable request, subject to institutional requirements and participant-privacy protections; network-address metadata are excluded from shared materials.

## Results

### Measurement quality

Cronbach's alpha ranged from 0.730 for interpersonal interaction to 0.837 for parasocial interaction, and McDonald's omega ranged from 0.744 for community identification to 0.838 for parasocial interaction. Standardized item loadings ranged from 0.436 to 0.804. The lowest loadings occurred for two parasocial interaction items, while all four self-efficacy loadings were close to 0.69. Composite reliability was above 0.74 for every construct. Average variance extracted ranged from 0.345 to 0.482 and remained below 0.50 for all constructs, although self-efficacy approached that level. The measures therefore showed adequate internal consistency but limited convergent validity at the item level (Table 2).

**Table 2:** Descriptive statistics and measurement quality.

Variable	Items	*M*	SD	Alpha	Omega	CR	AVE	Loading range
Parasocial interaction	10	3.845	0.585	0.837	0.838	0.838	0.345	0.436–0.683
Interpersonal interaction	4	3.914	0.658	0.730	0.757	0.757	0.444	0.518–0.804
Community identification	4	3.653	0.745	0.733	0.744	0.744	0.423	0.540–0.728
Self-efficacy	4	3.629	0.749	0.787	0.788	0.788	0.482	0.689–0.697
Customer value co-creation behavior	8	3.849	0.625	0.828	0.830	0.830	0.382	0.493–0.685

Alpha, Cronbach's alpha; Omega, McDonald's omega; CR, composite reliability; AVE, average variance extracted. Loading ranges are standardized estimates from the five-factor CFA.

The correlated five-factor model yielded chi-square(395) = 784.76, *p* < 0.001, CFI = 0.870, TLI = 0.857, RMSEA = 0.064, and SRMR = 0.056. RMSEA and SRMR were within commonly used ranges for approximate fit, whereas CFI and TLI were below conventional 0.90 criteria. The model should consequently be described as partially rather than clearly supported. It nevertheless fit significantly better than the model combining the two interaction constructs, delta chi-square(4) = 37.57, *p* < 0.001; the model combining community identification and self-efficacy, delta chi-square(4) = 16.05, *p* = 0.003; and the one-factor model, delta chi-square(10) = 91.06, *p* < 0.001 (Table 3).

**Table 3:** Confirmatory measurement model comparisons.

Model	Chi-square	df	CFI	TLI	RMSEA	SRMR	BIC
Five correlated factors	784.76	395	0.870	0.857	0.064	0.056	1168.40
Combined interaction factor	822.33	399	0.859	0.846	0.067	0.055	1184.05
Combined mediator factor	800.81	399	0.866	0.854	0.065	0.056	1162.53
One factor	875.81	405	0.843	0.831	0.070	0.056	1204.65
Five traits plus equal-loading method factor	705.06	394	0.896	0.885	0.057	0.062	1094.18

*N* = 240. The common method factor was constrained to be orthogonal to the five trait factors and to have equal loadings. The table reports relative BIC values calculated on the same covariance basis; lower values indicate better relative fit.

Discriminant-validity results were mixed. HTMT estimates ranged from 0.732 to 0.971. The ratios for parasocial interaction with interpersonal interaction, community identification, and customer value co-creation behavior exceeded 0.90, and their bootstrap intervals approached or included 1.00. Community identification also showed high HTMT values with customer value co-creation behavior and self-efficacy. The five-construct specification was empirically preferable to the collapsed alternatives, but several constructs were not sharply separated. This result is important for the structural analysis because unique regression paths may be unstable when predictors contain overlapping relational or evaluative content.

The equal-loading common method factor improved fit relative to the five-trait model, delta chi-square(1) = 79.70, *p* < 0.001, although CFI = 0.896 and TLI = 0.885 still did not indicate strong fit. The estimated common loading was 0.553. This sensitivity analysis is consistent with non-trivial shared-method covariance; it does not identify its exact size or remove it from the regression estimates.

The one-factor model for the eight Customer value co-creation items fit reasonably, chi-square(20) = 36.54, CFI = 0.968, TLI = 0.956, RMSEA = 0.059, SRMR = 0.039. The correlated participation-citizenship model yielded chi-square(19) = 33.94, CFI = 0.971, TLI = 0.958, RMSEA = 0.057, and SRMR = 0.039. The improvement was not statistically detectable, delta chi-square(1) = 2.60, *p* = 0.107, and BIC favored the one-factor model. These results support use of the overall composite for the primary analysis, while separate-outcome analyses remain informative because the two dimensions can have different antecedent patterns.

### Descriptive statistics and correlations

Mean scores were above the scale midpoint for all five variables. Interpersonal interaction had the highest mean (*M* = 3.914, SD = 0.658), followed by customer value co-creation behavior (*M* = 3.849, SD = 0.625), parasocial interaction (*M* = 3.845, SD = 0.585), community identification (*M* = 3.653, SD = 0.745), and self-efficacy (*M* = 3.629, SD = 0.749). All bivariate correlations were positive and statistically significant. Parasocial interaction correlated strongly with interpersonal interaction (*r* = 0.746), community identification (*r* = 0.765), self-efficacy (*r* = 0.717), and customer value co-creation behavior (*r* = 0.785). Interpersonal interaction correlated with community identification at *r* = 0.567, self-efficacy at *r* = 0.560, and customer value co-creation behavior at *r* = 0.677. Community identification and self-efficacy correlated at *r* = 0.692 (Table 4).

**Table 4:** Correlations among focal variables.

Variable	1	2	3	4	5
1Parasocial interaction	1.000				
2Interpersonal interaction	0.746^***^	1.000			
3 Community identification	0.765^***^	0.567^***^	1.000		
4 Self-efficacy	0.717^***^	0.560^***^	0.692^***^	1.000	
5 Customer value co-creation behavior	0.785^***^	0.677^***^	0.715^***^	0.709^***^	1.000

*N* = 240. ^***^p < 0.001.

These correlations show that both interaction variables were positively associated with both proposed mediators at the bivariate level. They also show why simultaneous estimation matters. A positive bivariate association may not remain unique after the substantial shared variance between parasocial interaction and interpersonal interaction is modeled.

### Structural paths

In the simultaneous model for community identification, parasocial interaction was positively associated with identification, *B* = 0.984, HC3 SE = 0.075, 95% CI [0.837, 1.132], standardized beta = 0.772, *p* < 0.001. Interpersonal interaction did not have a unique association, *B* = −0.011, HC3 SE = 0.070, 95% CI [−0.149, 0.128], standardized beta = −0.009, *p* = 0.881. The model explained 58.6% of the variance in community identification. H1a was supported, whereas H1b was not supported.

The corresponding model for self-efficacy produced a positive coefficient for parasocial interaction, *B* = 0.864, HC3 SE = 0.081, 95% CI [0.704, 1.025], standardized beta = 0.675, *p* < 0.001. The unique coefficient for interpersonal interaction was not significant, *B* = 0.064, HC3 SE = 0.074, 95% CI [−0.083, 0.210], standardized beta = 0.056, *p* = 0.393. The model explained 51.5% of the variance in self-efficacy. H4a was supported, and H4b was not supported.

In the total-association model, both interaction variables were positively associated with customer value co-creation behavior. Parasocial interaction had *B* = 0.676, HC3 SE = 0.069, 95% CI [0.540, 0.813], standardized beta = 0.633, *p* < 0.001. Interpersonal interaction had *B* = 0.194, HC3 SE = 0.058, 95% CI [0.079, 0.309], standardized beta = 0.204, *p* = 0.001. The model explained 63.5% of the outcome variance.

When community identification and self-efficacy were added, both remained positively associated with customer value co-creation behavior. The coefficient for community identification was *B* = 0.165, HC3 SE = 0.070, 95% CI [0.028, 0.303], standardized beta = 0.197, *p* = 0.018. The coefficient for self-efficacy was *B* = 0.193, HC3 SE = 0.069, 95% CI [0.058, 0.329], standardized beta = 0.232, *p* = 0.005. H2 and H5 were supported. Parasocial interaction retained a direct association, *B* = 0.346, 95% CI [0.163, 0.530], standardized beta = 0.324, *p* < 0.001, and interpersonal interaction also retained a direct association, *B* = 0.183, 95% CI [0.074, 0.292], standardized beta = 0.193, *p* = 0.001. The complete model explained 69.1% of the variance in customer value co-creation behavior (Table 5).

**Table 5:** Regression paths in the primary composite model.

Outcome and predictor	*B*	HC3 SE	95% CI	Standardized beta	*p*
Community identification					
Parasocial interaction	0.984	0.075	[.837, 1.132]	0.772	<0.001
Interpersonal interaction	−0.011	0.070	[-0.149,0.128]	−0.009	0.881
Self-efficacy					
Parasocial interaction	0.864	0.081	[0.704, 1.025]	0.675	<0.001
Interpersonal interaction	0.064	0.074	[−0.083, 0.210]	0.056	0.393
Customer value co-creation behavior with mediators					
Parasocial interaction	0.346	0.093	[0.163, 0.530]	0.324	<0.001
Interpersonal interaction	0.183	0.055	[0.074, 0.292]	0.193	0.001
Community identification	0.165	0.070	[0.028, 0.303]	0.197	0.018
Self-efficacy	0.193	0.069	[0.058, 0.329]	0.232	0.005

*N* = 240. Primary models are unadjusted. R-squared = 0.586 for community identification, 0.515 for self-efficacy, and 0.691 for customer value co-creation behavior with both mediators.

### Indirect associations

The specific indirect association between parasocial interaction and customer value co-creation behavior through community identification was *B* = 0.163, bootstrap SE = 0.068, 95% CI [0.038, 0.303]. The specific indirect association through self-efficacy was *B* = 0.167, bootstrap SE = 0.058, 95% CI [0.053, 0.281]. Both intervals excluded zero, supporting H3a and H6a. Their contrast was *B* = −0.005, 95% CI [−0.213, 0.214]. The analysis therefore did not detect a difference between the two indirect associations, but this result does not establish equivalence.

The indirect association of interpersonal interaction through community identification was *B* = −0.002, bootstrap SE = 0.012, 95% CI [−0.026, 0.025]. Its indirect association through self-efficacy was *B* = 0.012, bootstrap SE = 0.016, 95% CI [−0.013, 0.048]. Both intervals included zero. H3b and H6b were not supported. The total indirect association for interpersonal interaction was also not statistically detectable, *B* = 0.011, 95% CI [−0.030, 0.057]. Interpersonal interaction nevertheless retained a positive direct association with Customer value co-creation in the outcome model (Table 6).

**Table 6:** Bootstrap indirect associations.

Predictor	Statistical mediator	Indirect B	Bootstrap SE	95 percent bootstrap CI	Result
Parasocial interaction	Community identification	0.163	0.068	[0.038, 0.303]	Detectable
Parasocial interaction	Self-efficacy	0.167	0.058	[0.053, 0.281]	Detectable
Interpersonal interaction	Community identification	−0.002	0.012	[−0.026, 0.025]	Not detectable
Interpersonal interaction	Self-efficacy	0.012	0.016	[−0.013, 0.048]	Not detectable
Parasocial interaction	Total indirect	0.330	0.065	[0.205, 0.455]	Detectable
Interpersonal interaction	Total indirect	0.011	0.022	[−0.030, 0.057]	Not detectable
Parasocial interaction	Difference between mediators	−0.005	0.109	[−0.213, 0.214]	Not detectable
Interpersonal interaction	Difference between mediators	−0.014	0.018	[−0.050, 0.019]	Not detectable

*N* = 240. Results use 5,000 percentile-bootstrap samples. Detectable means that the 95% interval excludes zero; it does not establish temporal causality.

### Diagnostics and sensitivity analyses

The outcome model showed no evidence of functional-form misspecification in the Ramsey RESET test, *F* = 0.58, *p* = 0.558. Variance inflation factors ranged from 2.26 to 4.08, below a conventional threshold of 5 but consistent with meaningful shared variance. The Breusch-Pagan test indicated heteroskedasticity, chi-square(4) = 24.48, *p* < 0.001, which motivated HC3 standard errors. Residuals were non-normal in the Jarque-Bera test, *p* < 0.001. The maximum Cook's distance was 0.130; 22 observations exceeded the heuristic 4/N value, but none approached 1. The maximum leverage was 0.110. No observation was removed on the basis of these *post hoc* diagnostics.

Excluding the 15 uniform responders produced *N* = 225 and the same inferential pattern. The two parasocial interaction indirect associations remained statistically detectable through community identification, *B* = 0.159, 95% CI [0.035, 0.291], and self-efficacy, *B* = 0.157, 95% CI [0.044, 0.265]. The two interpersonal interaction indirect intervals continued to include zero. The outcome model coefficients for parasocial interaction, interpersonal interaction, community identification, and self-efficacy remained positive, and R-squared was 0.598. The lower R-squared relative to the full sample shows that uniform-response exclusion changes effect magnitude and should not be treated as inconsequential.

Adding dummy-coded demographic controls did not change the pattern. The parasocial interaction indirect associations were *B* = 0.167 through community identification and *B* = 0.184 through self-efficacy, with both intervals excluding zero. The two interpersonal interaction indirect intervals included zero. Adding duration and visit frequency produced the same conclusion. These sensitivity models explained 70.6% and 72.4% of outcome variance, respectively. Complete coefficients, HC3 robust standard errors, confidence intervals, *p* values, and reference categories appear in Appendix C (Tables 12 through 17); indirect estimates are summarized in Table 7.

**Table 7:** Sensitivity of indirect associations.

Model	*N*	PSI through CI	PSI through SE	II through CI	II through SE
Full sample primary	240	0.163 [0.0038, 0.303]	0.167 [0.0053, 0.281]	−0.002 [−0.026, 0.025]	0.012 [−0.013, 0.048]
Exclude uniform responders	225	0.159 [0.035, 0.291]	0.157 [0.044, 0.265]	−0.002 [-0.027, 0.026]	0.011 [−0.017, 0.049]
Demographic dummy controls	240	0.167 [0.035, 0.316]	0.184 [0.070, 0.300]	−0.004 [−0.029, 0.021]	0.017 [−0.012, 0.059]
Demographic and community-use controls	240	0.170 [0.040, 0.310]	0.186 [0.071, 0.296]	−0.007 [−0.034, 0.020]	0.015 [−0.014, 0.057]

Entries are unstandardized indirect associations with percentile 95% bootstrap intervals. PSI, parasocial interaction; II, interpersonal interaction; CI, community identification; SE, self-efficacy.

A simulation-based sensitivity analysis used the observed covariance structure and N = 240. The estimated detection probabilities were 0.915 and 0.986 for the two observed parasocial interaction indirect associations. The corresponding probabilities for the near-zero interpersonal interaction pathways were 0.020 and 0.077. These values describe precision under the observed parameterization; they do not convert non-significant effects into evidence of absence and should not be treated as retrospective confirmation of the study design.

When the outcome was separated, the total indirect association of parasocial interaction remained detectable for both participation behavior, *B* = 0.350, 95% CI [0.191, 0.496], and citizenship behavior, *B* = 0.310, 95% CI [0.175, 0.449]. The specific pattern differed: community identification accounted for a statistically detectable indirect association with participation, while self-efficacy accounted for a statistically detectable indirect association with citizenship. The other specific intervals narrowly or clearly included zero. Neither interpersonal interaction total indirect association was statistically detectable. This supplementary result shows that aggregating the two outcome dimensions preserves the overall parasocial interaction indirect association but can conceal differences between specific forms of co-creation (Table 8).

**Table 8:** Separate participation and citizenship outcomes.

Outcome	PSI through CI	PSI through SE	II through CI	II through SE
Participation behavior	0.224 [0.062, 0.380]	0.126 [−0.004, 0.261]	−0.002 [−0.034, 0.034]	0.009 [−0.011, 0.046]
Citizenship behavior	0.102 [−0.032, 0.254]	0.208 [0.070, 0.341]	−0.001 [−0.019, 0.018]	0.015 [−0.019, 0.061]

Entries are unstandardized indirect associations with percentile 95% bootstrap intervals. *N* = 240.

Table 9 summarizes the hypothesis tests. Six hypotheses were supported and four were not supported. The unsupported hypotheses all concerned the unique paths from interpersonal interaction to the proposed mediators after parasocial interaction was included.

**Table 9:** Summary of hypothesis tests.

Hypothesis	Proposed relationship	Result
H1a	Parasocial interaction positively predicts community identification	Supported
H1b	Interpersonal interaction positively predicts community identification	Not supported
H2	Community identification positively predicts customer value co-creation behavior	Supported
H3a	Community identification mediates the parasocial interaction association	Supported as a cross-sectional indirect association
H3b	Community identification mediates the interpersonal interaction association	Not supported
H4a	Parasocial interaction positively predicts self-efficacy	Supported
H4b	Interpersonal interaction positively predicts self-efficacy	Not supported
H5	Self-efficacy positively predicts customer value co-creation behavior	Supported
H6a	Self-efficacy mediates the parasocial interaction association	Supported as a cross-sectional indirect association
H6b	Self-efficacy mediates the interpersonal interaction association	Not supported

## Discussion

### Main findings

Parasocial interaction was uniquely associated with community identification and self-efficacy, and both variables were independently associated with customer value co-creation behavior. The two corresponding bootstrap indirect associations were statistically detectable. Interpersonal interaction showed positive bivariate correlations with both mediators and with co-creation, but its unique coefficients for the mediators were close to zero after parasocial interaction was included. It retained a direct association with co-creation. The evidence therefore supports two indirect associations for parasocial interaction, not a general claim that both interaction sources operate through both psychological variables.

The result should not be interpreted as proof that brand-side interaction is more important than peer interaction. Parasocial interaction and interpersonal interaction correlated at *r* = 0.746, the latent factor correlation was even higher, and the HTMT ratio exceeded 0.90. The two measures may capture overlapping community positivity, engagement, or social presence in addition to source-specific interaction. Under this degree of overlap, regression coefficients represent the remaining unique part of each composite, not the total practical relevance of each interaction source. Interpersonal interaction also retained a positive direct coefficient in the outcome equation, which is compatible with pathways not represented by community identification or self-efficacy, such as immediate information exchange, reciprocal problem solving, or direct opportunities to contribute.

### Community identification and self-efficacy

Community identification was positively associated with Customer value co-creation after the other variables were held constant. This result is consistent with social identity theory and prior virtual-community research (Luo et al., 2016; Kaur et al., 2020; Liu et al., 2024). Members who view the community as part of the self may attach identity-relevant meaning to feedback, helping, and advocacy. In the present data, community identification statistically accounted for part of the parasocial interaction-co-creation association.

Self-efficacy also retained an independent association with Customer value co-creation. This finding is consistent with social cognitive theory and with research linking context-specific efficacy beliefs to knowledge sharing and co-creation (Hsu et al., 2007; Alves and Mainardes, 2017; Hasan et al., 2024). Members who believe that they can use community functions, express ideas, share experience, and answer questions may face a lower psychological threshold for active participation. Self-efficacy statistically accounted for a second part of the parasocial interaction-co-creation association.

The contrast between these two indirect associations included zero. The appropriate conclusion is that the study did not detect a difference, not that the pathways are equal. The separate-outcome analyses further qualify the comparison. The identification path was clearer for participation behavior, whereas the efficacy path was clearer for citizenship behavior. These patterns require replication because they were supplementary, but they show why the two mediators and two outcome dimensions should not be reduced to a claim of equal complementary mechanisms.

### Interpretation of parasocial interaction

The parasocial interaction scale requires a bounded interpretation. Its items include friend-like experience and willingness to approach the brand, but they also include trustworthiness, friendliness, positive affect, information sharing, and perceived value of attention. The construct is therefore broader than a momentary parasocial interaction with an identifiable media persona. It is closer to a relational perception of the brand side within a mediated community. That breadth may explain its strong correlations with community identification, self-efficacy, and Customer value co-creation.

Contemporary social media research supports caution about a strict one-way vs. two-way distinction. Social presence, self-disclosure, visible responsiveness, and intermittent reciprocal contact can coexist with audience-like relationships (Kim, 2022; Lou, 2022; Balaban et al., 2022). In a virtual brand community, members may respond to an official account as a social actor even when individual attention is limited. The present results are consistent with relational brand-side experience covarying with both belonging and perceived capability, but they do not show which platform cue produced that perception. Future measures should distinguish the brand, official account, spokesperson, and community manager as targets and record whether actual direct responses occurred.

### Theoretical implications

The study has several theoretical contributions. First, it shows that source-specific interaction variables do not necessarily retain symmetrical psychological pathways when estimated together. Positive bivariate associations for peer interaction did not become unique identification or self-efficacy paths after the overlapping brand-side relational measure was included. A model that treats all interaction as interchangeable would miss this suppression by shared variance.

Second, the study distinguishes relational and capability-related organismic variables without claiming that the SOR classification alone is a theoretical advance. Social identity theory explains why community membership can become self-relevant. Social cognitive theory explains why social cues can relate to beliefs about contribution capability. Their inclusion in one model permits a test of unique covariance, but it does not establish that the variables operate in parallel over time. Identification could influence efficacy through access to support, efficacy could influence identification through successful contribution, and both could be outcomes of a more general positive community evaluation.

Third, the measurement results expose a theoretical boundary. The five-factor model fit better than collapsed alternatives, but absolute fit and discriminant validity were incomplete. The distinction between parasocial and interpersonal interaction is therefore conceptually useful but empirically fragile in this instrument. Future theory should specify whether the key difference is interaction target, degree of reciprocity, social presence, or perceived relational closeness. Better measures may show that these dimensions cross the simple brand-vs.-peer division.

Fourth, the analysis refines the interpretation of customer value co-creation. The one-factor model was adequate, but the participation and citizenship analyses did not produce identical specific indirect patterns. Yi and Gong (2013) distinguished in-role participation from voluntary citizenship, and later research continues to treat these forms as potentially different behavioral outcomes. Models of virtual brand communities should therefore justify aggregation and, where possible, report both levels.

### Practical implications

The findings provide cautious guidance for community design. Brand-side communication should make contribution procedures understandable and should show how member input is received. Clear instructions, visible responses, examples of adopted suggestions, and feedback about outcomes can support both relational recognition and contribution confidence. These practices are consistent with the observed associations of parasocial interaction with community identification and self-efficacy, although the cross-sectional design does not show that implementing them will cause those changes.

Peer interaction remains practically relevant even though its proposed indirect paths were not unique. Interpersonal interaction was positively correlated with every focal variable and retained a direct association with Customer value co-creation. Community operators can reduce friction in member-to-member exchange through searchable questions, experience tags, mutual-help functions, and recognition for useful answers. The results do not justify replacing peer features with brand communication. They instead suggest that peer exchange may relate to co-creation through immediate behavioral opportunities or other psychological variables not measured here.

Community managers should avoid using interaction counts as the only success criterion. A high number of posts can reflect activity without clarifying whether members feel recognized, capable, or able to make a useful contribution. Short onboarding tasks, contribution templates, examples of high-quality feedback, and closed-loop reports on member suggestions can make participation requirements more visible. Separate metrics for participation and citizenship may also be useful because information provision, advocacy, helping, and tolerance need not respond to the same community conditions.

### Limitations and future research

The most important limitation is temporal. All variables were measured from the same respondents at one time. A significant cross-sectional indirect effect shows that the covariance pattern is compatible with the specified equations; it does not establish the sequence Interaction to mediator to behavior. Cross-sectional estimates can be biased representations of longitudinal mediation even when a causal process exists (Maxwell and Cole, 2007). Reverse and reciprocal relationships are plausible. Active contributors may receive more brand responses, successful contribution may increase self-efficacy, and continued participation may strengthen identification. A three-wave design separating interaction, psychological variables, and behavior would better test temporal order. Experiments could manipulate brand responsiveness or peer-support visibility while holding content constant.

Second, all focal variables were measured by self-report in one questionnaire. The common method factor sensitivity analysis indicated non-trivial shared covariance. The analysis cannot determine whether this covariance reflects acquiescence, social desirability, halo evaluation, item similarity, or a substantive general community attitude. Future studies should combine surveys with behavioral records such as posts, replies, accepted suggestions, helping episodes, or participation in product-testing activities. Temporal separation and multiple sources would also reduce dependence on a single response context.

Third, measurement validity was only partly supported. Reliability was acceptable, but AVE values were below 0.50, CFI and TLI were below 0.90, and several HTMT values were high. The parasocial interaction measure includes broad evaluative and relational content and should not be treated as a pure indicator of classic parasocial interaction. The structural estimates are therefore exploratory composite associations subject to measurement overlap. Future work should use independently validated, target-specific scales and should distinguish perceived interactivity, social presence, brand anthropomorphism, brand responsiveness, parasocial experience, and actual reciprocal communication.

Fourth, the sampling method limits generalization. The sample was obtained through snowball distribution in China and was concentrated among adults aged 18 to 30 and respondents with a bachelor's degree. Although this demographic profile may partly resemble that of highly active virtual brand community users, it restricts generalizability. The study did not record the specific community or platform for each participant. The findings cannot be assumed to generalize to older users, other cultural settings, or specialized communities. Community affordances differ across gaming, beauty, automotive, technology, financial, and professional-service contexts. Future research should record platform, community category, community size, and respondent nesting so that contextual variation and clustered observations can be modeled.

Fifth, data-quality information was limited. The raw export contained completion time and network metadata but no attention check or validated bot indicator. Uniform responding was not logically invalid because all items were positively keyed, so the full sample was retained and exclusion was treated as sensitivity analysis. Future questionnaires should include balanced item wording, instructed-response checks, and planned timing rules. Any exclusion rule should be defined before outcome analysis.

Sixth, the hypotheses and model were not preregistered and were developed after the dataset had been collected. The study should therefore be viewed as theory-guided secondary analysis, not a confirmatory test in the strict sense. Replication with preregistered measurement composition, exclusion rules, and analysis code is necessary. The present article makes the corrected mapping and null hypotheses visible so that later studies can test the revised pattern without selective reporting.

## Conclusion

This study examined associations among parasocial interaction, interpersonal interaction, community identification, self-efficacy, and customer value co-creation behavior in a cross-sectional survey of virtual brand community users. The five measures showed acceptable internal consistency but only partial measurement-model support. Parasocial interaction had statistically detectable indirect associations with Customer value co-creation through both community identification and self-efficacy. Interpersonal interaction did not uniquely predict either proposed mediator after parasocial interaction was included, although it retained a direct association with Customer value co-creation.

The results therefore do not support a symmetrical four-path account. They suggest that brand-side relational experience and peer interaction share substantial variance but may relate to co-creation in different ways. Community identification and self-efficacy remain useful relational and capability-related correlates, yet the data cannot establish their causal or temporal role. Stronger inference will require target-specific measurement, longitudinal or experimental designs, behavioral outcomes, and community-level context.

## Data Availability

The raw data supporting the conclusions of this article will be made available by the authors, without undue reservation.
