# Supplementary material for: Parasocial and interpersonal interaction: distinct associations with customer value co-creation in virtual brand communities
Source: Front Psychol. 2026 Oct 21;17:1975666. doi: 10.3389/fpsyg.2026.1975666 (PMC13731342; doi:10.3389/fpsyg.2026.1975666)
Supplement: Supplementary file 1 [file Supplementary_file_1.docx]

## Appendix A Measurement items

**Table 10 Complete item wording and construct assignment**

| **Code** | **Variable** | **English wording** | **Chinese questionnaire wording** | **Primary source** |
| --- | --- | --- | --- | --- |
| PSI1 | Parasocial interaction | I find this brand community attractive | 我觉得这个品牌社区很有吸引力 | Bao et al. 2011 and Hoerner 1999 |
| PSI2 | Parasocial interaction | I would like to continue following information published by this brand community | 我会持续关注该品牌社区发布的各类信息 | Bao et al. 2011 and Hoerner 1999 |
| PSI3 | Parasocial interaction | I would like to ask this brand community questions | 我愿意在该品牌社区向品牌咨询相关问题 | Bao et al. 2011 and Hoerner 1999 |
| PSI4 | Parasocial interaction | Interacting with the brand feels like being with a good friend | 与该品牌互动，就像和一位好朋友相处 | Bao et al. 2011 and Hoerner 1999 |
| PSI5 | Parasocial interaction | I feel that this brand is trustworthy | 我感觉这个品牌是可信的 | Bao et al. 2011 and Hoerner 1999 |
| PSI6 | Parasocial interaction | This brand can help me achieve my goals | 这个品牌能够帮助我实现目标 | Bao et al. 2011 and Hoerner 1999 |
| PSI7 | Parasocial interaction | I am willing to share information about this brand with my friends | 我愿意把该品牌的相关信息分享给我的朋友 | Bao et al. 2011 and Hoerner 1999 |
| PSI8 | Parasocial interaction | I feel relaxed and pleasant when browsing information in this brand community | 在浏览该品牌社区信息时，我感到放松愉悦 | Bao et al. 2011 and Hoerner 1999 |
| PSI9 | Parasocial interaction | I think this brand is friendly | 我认为该品牌是友好的 | Bao et al. 2011 and Hoerner 1999 |
| PSI10 | Parasocial interaction | I think spending time following this brand is worthwhile | 我认为花时间关注这个品牌是值得的 | Bao et al. 2011 and Hoerner 1999 |
| II1 | Interpersonal interaction | In this brand community, other members and I exchange product-related information | 在该虚拟品牌社区中，我和其他社区成员会相互交流产品相关信息 | Wang and Ma 2013 |
| II2 | Interpersonal interaction | In this brand community, other members and I share product-use experience | 在该虚拟品牌社区中，我和其他社区成员会互相分享使用体验 | Wang and Ma 2013 |
| II3 | Interpersonal interaction | In this brand community, other members and I engage in emotional exchange | 在该虚拟品牌社区中，我和其他社区成员会进行情感交流 | Wang and Ma 2013 |
| II4 | Interpersonal interaction | In this brand community, other members and I give each other advice | 在该虚拟品牌社区中，我和其他社区成员会互相给予建议 | Wang and Ma 2013 |
| CI1 | Community identification | I see myself as a member of this virtual brand community | 我认为自己是这个虚拟品牌社区的一员 | Algesheimer et al. 2005 |
| CI2 | Community identification | I am proud to be a member of this virtual brand community | 我为自己是该虚拟品牌社区的成员而感到自豪 | Algesheimer et al. 2005 |
| CI3 | Community identification | I feel a strong sense of belonging to this virtual brand community | 我对这个虚拟品牌社区有强烈的归属感 | Algesheimer et al. 2005 |
| CI4 | Community identification | Joining this virtual brand community enables me to gain respect from others | 加入虚拟品牌社区能够让我获得他人的尊重 | Algesheimer et al. 2005 |
| VCC1 | Customer value co-creation behavior participation | I actively obtain brand or product information from the community | 我主动从社区获取品牌/产品相关信息 | Yi and Gong 2013 |
| VCC2 | Customer value co-creation behavior participation | I provide information that the brand needs | 我向社区提供品牌方需要的相关信息 | Yi and Gong 2013 |
| VCC3 | Customer value co-creation behavior participation | I fulfill my responsibilities as a community member | 我履行作为社区成员应尽的责任 | Yi and Gong 2013 |
| VCC4 | Customer value co-creation behavior participation | I can communicate effectively with community operators | 我能和社区运营人员有效沟通 | Yi and Gong 2013 |
| VCC5 | Customer value co-creation behavior citizenship | If I have suggestions for improving the community, I tell the community management | 如果我有改进社区的建议，我会告诉社区管理方 | Yi and Gong 2013 |
| VCC6 | Customer value co-creation behavior citizenship | I recommend this virtual brand community to others | 我会向他人推荐这个虚拟品牌社区 | Yi and Gong 2013 |
| VCC7 | Customer value co-creation behavior citizenship | I help other community members when they have problems | 如果其他社区成员遇到问题，我会帮助他们 | Yi and Gong 2013 |
| VCC8 | Customer value co-creation behavior citizenship | I tolerate minor problems in the brand's products or services | 我会谅解该品牌的产品或服务中的瑕疵 | Yi and Gong 2013 |
| SE1 | Self-efficacy | Without guidance, I am confident that I can use the community's functions | 无需他人指导，我有信心使用该社区的各项功能 | Hsu et al. 2007 |
| SE2 | Self-efficacy | I am confident that I can express my views in this community | 我有信心在该社区表达我的观点 | Hsu et al. 2007 |
| SE3 | Self-efficacy | I believe that I can share product-use experience in this community | 我相信我能够在该社区分享产品使用体验 | Hsu et al. 2007 |
| SE4 | Self-efficacy | I am confident that I can answer questions from other community members | 我有信心回答社区其他成员提出的问题 | Hsu et al. 2007 |

Note. All items used response anchors from 1 strongly disagree to 5 strongly agree. No items were reverse coded.

## Appendix B Additional statistical information

**Table 11 Item diagnostics and model diagnostics**

| **Variable** | **Corrected item-total correlation range** | **Alpha if item deleted range** | **Additional diagnostic** |
| --- | --- | --- | --- |
| Parasocial interaction | .418-.623 | .813-.832 | Mean inter-item correlation = .339 |
| Interpersonal interaction | .400-.731 | .542-.732 | Mean inter-item correlation = .405 |
| Community identification | .391-.599 | .626-.740 | Mean inter-item correlation = .402 |
| Self-efficacy | .549-.633 | .716-.758 | Mean inter-item correlation = .485 |
| Customer value co-creation behavior | .418-.618 | .799-.828 | Mean inter-item correlation = .378 |

Note. Outcome-model diagnostics were maximum VIF = 4.08, Ramsey RESET p = .558, Breusch-Pagan p < .001, Jarque-Bera p < .001, maximum leverage = .110, and maximum Cook's distance = .130. Demographic-control reference categories were male, age 18 to 25 years, bachelor degree, no income, and student. Community-use reference categories were less than 6 months of participation and daily visits. Complete control-model coefficients are reported in Appendix C (Tables 12 through 17).

## Appendix C Complete control model coefficients

Tables 12 through 17 report every coefficient from the demographic-control and demographic-and-community-use sensitivity models. The model equations otherwise match the primary composite analysis.

**Table 12 Demographic-control model predicting community identification**

| **Predictor** | **B** | **HC3 SE** | **95% CI** | **p** |
| --- | --- | --- | --- | --- |
| Intercept | -.165 | .324 | [-.804, .473] | .610 |
| Parasocial interaction | 1.014 | .079 | [.859, 1.169] | <.001 |
| Interpersonal interaction | -.026 | .074 | [-.172, .119] | .722 |
| Gender: female | -.019 | .069 | [-.154, .116] | .781 |
| Age: 26 to 30 years | .135 | .214 | [-.286, .557] | .528 |
| Age: 31 to 35 years | .045 | .210 | [-.368, .458] | .829 |
| Age: over 35 years | .097 | .218 | [-.334, .527] | .659 |
| Education: high school or below | .144 | .139 | [-.130, .418] | .302 |
| Education: associate degree | -.039 | .210 | [-.453, .376] | .855 |
| Education: master degree | .126 | .081 | [-.034, .287] | .122 |
| Education: doctorate or above | -.143 | .488 | [-1.104, .819] | .770 |
| Income: below RMB 2,000 | .155 | .107 | [-.057, .366] | .152 |
| Income: RMB 2,000 to 5,000 | .118 | .160 | [-.198, .434] | .462 |
| Income: RMB 5,001 to 10,000 | .019 | .171 | [-.318, .356] | .912 |
| Income: RMB 10,001 to 30,000 | .131 | .183 | [-.231, .493] | .476 |
| Income: RMB 30,001 to 50,000 | .025 | .243 | [-.454, .504] | .918 |
| Income: above RMB 50,000 | -.201 | .275 | [-.743, .340] | .465 |
| Occupation: teacher | -.155 | .423 | [-.989, .680] | .715 |
| Occupation: government or public institution | -.261 | .218 | [-.692, .169] | .233 |
| Occupation: corporate employee | -.163 | .162 | [-.482, .157] | .316 |
| Occupation: self-employed | -.004 | .228 | [-.453, .446] | .987 |
| Occupation: other | -.388 | .260 | [-.899, .123] | .136 |

Note. N = 240. R-squared = 0.631. Coefficients are unstandardized; confidence intervals and p values use HC3 robust standard errors. Reference categories were male, age 18 to 25 years, bachelor degree, no income, and student.

**Table 13 Demographic-control model predicting self-efficacy**

| **Predictor** | **B** | **HC3 SE** | **95% CI** | **p** |
| --- | --- | --- | --- | --- |
| Intercept | -.535 | .319 | [-1.163, .094] | .095 |
| Parasocial interaction | .903 | .084 | [.737, 1.069] | <.001 |
| Interpersonal interaction | .085 | .079 | [-.071, .240] | .284 |
| Gender: female | -.066 | .076 | [-.215, .083] | .381 |
| Age: 26 to 30 years | .456 | .233 | [-.004, .916] | .052 |
| Age: 31 to 35 years | .341 | .228 | [-.108, .790] | .136 |
| Age: over 35 years | .346 | .254 | [-.154, .846] | .174 |
| Education: high school or below | -.177 | .152 | [-.476, .123] | .246 |
| Education: associate degree | .279 | .165 | [-.046, .604] | .092 |
| Education: master degree | -.129 | .141 | [-.408, .150] | .364 |
| Education: doctorate or above | -.072 | .162 | [-.391, .247] | .658 |
| Income: below RMB 2,000 | .064 | .181 | [-.293, .420] | .726 |
| Income: RMB 2,000 to 5,000 | .108 | .161 | [-.208, .425] | .501 |
| Income: RMB 5,001 to 10,000 | .135 | .182 | [-.224, .494] | .460 |
| Income: RMB 10,001 to 30,000 | .250 | .189 | [-.123, .623] | .187 |
| Income: RMB 30,001 to 50,000 | .410 | .214 | [-.012, .832] | .057 |
| Income: above RMB 50,000 | .104 | .194 | [-.279, .486] | .594 |
| Occupation: teacher | -.009 | .241 | [-.484, .466] | .970 |
| Occupation: government or public institution | -.211 | .252 | [-.707, .284] | .402 |
| Occupation: corporate employee | -.166 | .186 | [-.531, .200] | .374 |
| Occupation: self-employed | .188 | .193 | [-.192, .568] | .331 |
| Occupation: other | .170 | .309 | [-.439, .779] | .583 |

Note. N = 240. R-squared = 0.561. Coefficients are unstandardized; confidence intervals and p values use HC3 robust standard errors. Reference categories were male, age 18 to 25 years, bachelor degree, no income, and student.

**Table 14 Demographic-control model predicting customer value co-creation behavior**

| **Predictor** | **B** | **HC3 SE** | **95% CI** | **p** |
| --- | --- | --- | --- | --- |
| Intercept | .356 | .251 | [-.139, .851] | .158 |
| Parasocial interaction | .330 | .101 | [.130, .530] | .001 |
| Interpersonal interaction | .186 | .060 | [.067, .305] | .002 |
| Community identification | .165 | .075 | [.018, .312] | .028 |
| Self-efficacy | .204 | .071 | [.064, .345] | .005 |
| Gender: female | -.029 | .055 | [-.138, .079] | .594 |
| Age: 26 to 30 years | .163 | .134 | [-.101, .427] | .225 |
| Age: 31 to 35 years | .228 | .130 | [-.028, .484] | .081 |
| Age: over 35 years | .176 | .137 | [-.093, .445] | .199 |
| Education: high school or below | -.018 | .103 | [-.221, .184] | .858 |
| Education: associate degree | -.176 | .133 | [-.438, .086] | .187 |
| Education: master degree | .038 | .080 | [-.120, .196] | .638 |
| Education: doctorate or above | .062 | .369 | [-.665, .790] | .866 |
| Income: below RMB 2,000 | -.033 | .116 | [-.261, .195] | .775 |
| Income: RMB 2,000 to 5,000 | -.076 | .141 | [-.354, .203] | .592 |
| Income: RMB 5,001 to 10,000 | -.123 | .139 | [-.396, .150] | .376 |
| Income: RMB 10,001 to 30,000 | -.086 | .144 | [-.370, .199] | .554 |
| Income: RMB 30,001 to 50,000 | -.225 | .281 | [-.780, .329] | .424 |
| Income: above RMB 50,000 | -.280 | .254 | [-.781, .221] | .272 |
| Occupation: teacher | .180 | .197 | [-.209, .568] | .363 |
| Occupation: government or public institution | .225 | .175 | [-.121, .570] | .201 |
| Occupation: corporate employee | .102 | .147 | [-.187, .391] | .489 |
| Occupation: self-employed | .086 | .188 | [-.286, .457] | .649 |
| Occupation: other | .118 | .221 | [-.317, .553] | .594 |

Note. N = 240. R-squared = 0.706. Coefficients are unstandardized; confidence intervals and p values use HC3 robust standard errors. Reference categories were male, age 18 to 25 years, bachelor degree, no income, and student.

**Table 15 Demographic and community-use control model predicting community identification**

| **Predictor** | **B** | **HC3 SE** | **95% CI** | **p** |
| --- | --- | --- | --- | --- |
| Intercept | -.175 | .387 | [-.939, .589] | .652 |
| Parasocial interaction | 1.004 | .084 | [.839, 1.170] | <.001 |
| Interpersonal interaction | -.040 | .076 | [-.189, .110] | .602 |
| Gender: female | -.021 | .070 | [-.158, .116] | .766 |
| Age: 26 to 30 years | .150 | .240 | [-.324, .624] | .533 |
| Age: 31 to 35 years | .050 | .233 | [-.409, .509] | .829 |
| Age: over 35 years | .097 | .242 | [-.381, .575] | .688 |
| Education: high school or below | .211 | .142 | [-.069, .492] | .138 |
| Education: associate degree | -.016 | .210 | [-.429, .397] | .939 |
| Education: master degree | .161 | .095 | [-.027, .349] | .092 |
| Education: doctorate or above | -.151 | .478 | [-1.094, .791] | .752 |
| Income: below RMB 2,000 | .166 | .125 | [-.081, .413] | .186 |
| Income: RMB 2,000 to 5,000 | .129 | .182 | [-.229, .488] | .477 |
| Income: RMB 5,001 to 10,000 | -.017 | .194 | [-.400, .365] | .929 |
| Income: RMB 10,001 to 30,000 | .077 | .201 | [-.319, .472] | .703 |
| Income: RMB 30,001 to 50,000 | -.064 | .338 | [-.729, .602] | .851 |
| Income: above RMB 50,000 | -.239 | .294 | [-.818, .341] | .417 |
| Occupation: teacher | -.198 | .460 | [-1.104, .709] | .668 |
| Occupation: government or public institution | -.287 | .240 | [-.760, .186] | .233 |
| Occupation: corporate employee | -.190 | .173 | [-.532, .152] | .274 |
| Occupation: self-employed | -.051 | .228 | [-.500, .399] | .825 |
| Occupation: other | -.426 | .297 | [-1.011, .158] | .152 |
| Participation duration: 6 months to 1 year | .029 | .113 | [-.195, .252] | .801 |
| Participation duration: 1 to 2 years | .171 | .115 | [-.057, .398] | .140 |
| Participation duration: 2 to 3 years | .316 | .147 | [.027, .605] | .033 |
| Participation duration: more than 3 years | .147 | .135 | [-.118, .413] | .276 |
| Visit frequency: every 2 to 4 days | -.020 | .088 | [-.193, .152] | .816 |
| Visit frequency: once a week | .033 | .114 | [-.192, .257] | .774 |
| Visit frequency: twice a month | -.127 | .253 | [-.626, .372] | .616 |
| Visit frequency: once a month | .356 | .320 | [-.275, .986] | .268 |
| Visit frequency: less than once a month | .038 | .172 | [-.301, .377] | .826 |

Note. N = 240. R-squared = 0.650. Coefficients are unstandardized; confidence intervals and p values use HC3 robust standard errors. Reference categories were male, age 18 to 25 years, bachelor degree, no income, student, less than 6 months of community participation, and daily visits.

**Table 16 Demographic and community-use control model predicting self-efficacy**

| **Predictor** | **B** | **HC3 SE** | **95% CI** | **p** |
| --- | --- | --- | --- | --- |
| Intercept | -.467 | .351 | [-1.159, .225] | .185 |
| Parasocial interaction | .886 | .088 | [.712, 1.059] | <.001 |
| Interpersonal interaction | .072 | .082 | [-.089, .234] | .378 |
| Gender: female | -.060 | .080 | [-.218, .097] | .451 |
| Age: 26 to 30 years | .435 | .243 | [-.043, .914] | .074 |
| Age: 31 to 35 years | .305 | .241 | [-.170, .781] | .207 |
| Age: over 35 years | .301 | .271 | [-.233, .835] | .268 |
| Education: high school or below | -.144 | .176 | [-.491, .203] | .413 |
| Education: associate degree | .312 | .173 | [-.029, .653] | .073 |
| Education: master degree | -.087 | .157 | [-.397, .223] | .579 |
| Education: doctorate or above | -.076 | .195 | [-.461, .309] | .698 |
| Income: below RMB 2,000 | .093 | .199 | [-.299, .485] | .640 |
| Income: RMB 2,000 to 5,000 | .119 | .181 | [-.238, .477] | .511 |
| Income: RMB 5,001 to 10,000 | .111 | .206 | [-.295, .516] | .591 |
| Income: RMB 10,001 to 30,000 | .200 | .212 | [-.218, .618] | .347 |
| Income: RMB 30,001 to 50,000 | .330 | .253 | [-.168, .828] | .193 |
| Income: above RMB 50,000 | .085 | .205 | [-.318, .489] | .677 |
| Occupation: teacher | -.051 | .280 | [-.603, .501] | .856 |
| Occupation: government or public institution | -.221 | .275 | [-.763, .320] | .421 |
| Occupation: corporate employee | -.169 | .206 | [-.575, .236] | .411 |
| Occupation: self-employed | .140 | .211 | [-.276, .556] | .508 |
| Occupation: other | .109 | .336 | [-.554, .772] | .746 |
| Participation duration: 6 months to 1 year | -.050 | .153 | [-.352, .251] | .742 |
| Participation duration: 1 to 2 years | .095 | .146 | [-.192, .383] | .514 |
| Participation duration: 2 to 3 years | .226 | .158 | [-.086, .538] | .155 |
| Participation duration: more than 3 years | .095 | .180 | [-.260, .450] | .600 |
| Visit frequency: every 2 to 4 days | .039 | .100 | [-.159, .236] | .702 |
| Visit frequency: once a week | .028 | .130 | [-.229, .285] | .831 |
| Visit frequency: twice a month | -.282 | .219 | [-.715, .150] | .199 |
| Visit frequency: once a month | .365 | .290 | [-.208, .937] | .211 |
| Visit frequency: less than once a month | .026 | .201 | [-.370, .422] | .896 |

Note. N = 240. R-squared = 0.582. Coefficients are unstandardized; confidence intervals and p values use HC3 robust standard errors. Reference categories were male, age 18 to 25 years, bachelor degree, no income, student, less than 6 months of community participation, and daily visits.

**Table 17 Demographic and community-use control model predicting customer value co-creation behavior**

| **Predictor** | **B** | **HC3 SE** | **95% CI** | **p** |
| --- | --- | --- | --- | --- |
| Intercept | .297 | .302 | [-.298, .892] | .326 |
| Parasocial interaction | .315 | .104 | [.111, .520] | .003 |
| Interpersonal interaction | .195 | .061 | [.075, .316] | .002 |
| Community identification | .169 | .076 | [.020, .318] | .026 |
| Self-efficacy | .210 | .072 | [.068, .351] | .004 |
| Gender: female | -.023 | .056 | [-.133, .088] | .685 |
| Age: 26 to 30 years | .128 | .140 | [-.148, .403] | .362 |
| Age: 31 to 35 years | .216 | .137 | [-.054, .485] | .116 |
| Age: over 35 years | .146 | .141 | [-.132, .424] | .301 |
| Education: high school or below | .007 | .119 | [-.227, .241] | .950 |
| Education: associate degree | -.155 | .146 | [-.442, .132] | .288 |
| Education: master degree | .026 | .080 | [-.132, .184] | .744 |
| Education: doctorate or above | .013 | .359 | [-.695, .720] | .972 |
| Income: below RMB 2,000 | -.030 | .125 | [-.277, .216] | .809 |
| Income: RMB 2,000 to 5,000 | -.076 | .146 | [-.365, .212] | .602 |
| Income: RMB 5,001 to 10,000 | -.124 | .147 | [-.414, .166] | .400 |
| Income: RMB 10,001 to 30,000 | -.086 | .155 | [-.391, .219] | .579 |
| Income: RMB 30,001 to 50,000 | -.214 | .319 | [-.843, .414] | .502 |
| Income: above RMB 50,000 | -.298 | .258 | [-.806, .211] | .250 |
| Occupation: teacher | .065 | .201 | [-.331, .461] | .745 |
| Occupation: government or public institution | .155 | .182 | [-.203, .513] | .396 |
| Occupation: corporate employee | .011 | .148 | [-.280, .302] | .941 |
| Occupation: self-employed | .007 | .198 | [-.383, .396] | .973 |
| Occupation: other | .150 | .229 | [-.302, .602] | .513 |
| Participation duration: 6 months to 1 year | .268 | .121 | [.030, .506] | .028 |
| Participation duration: 1 to 2 years | .150 | .124 | [-.094, .394] | .228 |
| Participation duration: 2 to 3 years | .167 | .128 | [-.085, .418] | .193 |
| Participation duration: more than 3 years | .189 | .130 | [-.067, .445] | .147 |
| Visit frequency: every 2 to 4 days | -.006 | .075 | [-.153, .141] | .941 |
| Visit frequency: once a week | -.131 | .082 | [-.294, .031] | .112 |
| Visit frequency: twice a month | -.041 | .092 | [-.223, .141] | .659 |
| Visit frequency: once a month | -.024 | .185 | [-.389, .342] | .898 |
| Visit frequency: less than once a month | .016 | .157 | [-.293, .325] | .919 |

Note. N = 240. R-squared = 0.724. Coefficients are unstandardized; confidence intervals and p values use HC3 robust standard errors. Reference categories were male, age 18 to 25 years, bachelor degree, no income, student, less than 6 months of community participation, and daily visits.
